# Association of isochromosome (7)(q10) in Shwachman–Diamond syndrome with the severity of cytopenia

**DOI:** 10.1002/ccr3.1249

**Published:** 2017-12-05

**Authors:** Yuko Shimosato, Reo Tanoshima, Shin‐ichi Tsujimoto, Masanobu Takeuchi, Koji Sasaki, Ryosuke Kajiwara, Hiroaki Goto, Junichi Nagai, Masakatsu D. Yanagimachi, Shuichi Ito, Shumpei Yokota

**Affiliations:** ^1^ Department of Pediatrics Yokohama City University School of Medicine Yokohama Japan; ^2^ Kanagawa Children's Medical Center Yokohama Japan

**Keywords:** cytopenia, i(7q), Shwachman–Diamond syndrome

## Abstract

We report two male siblings with SDS. They have the same compound heterozygous mutations. Only one of the siblings acquired cytogenetic abnormality of i(7q) 2 years after diagnosis, became transfusion‐dependent, and underwent allogeneic hematopoietic stem cell transplantation. These cases indicate that i(7q) is associated with significant cytopenia in SDS patients.

## Introduction

Shwachman–Diamond syndrome (SDS) (OMIM: #260400) is an autosomal recessive disorder characterized by skeletal anomalies, exocrine pancreatic dysfunction, and bone marrow failure 1, 2. Also associated with SDS are blood disorders such as cytopenia, myelodysplastic syndrome, and acute myeloid leukemia. Hematopoietic cell transplantation (HCT) is the only curative treatment for the severe bone marrow failure associated with SDS 3.

Somatic mutations of the Shwachman–Bodian–Diamond syndrome (SBDS) gene were identified in 90% of patients with SDS 2, 4. Correlations between specific SBDS gene mutations and the severity of the disease have not been elucidated 1, 5, 6.

Besides SBDS gene mutations, cytogenetic abnormalities of bone marrow have been identified in some SDS patients with bone marrow failure. However, none of those abnormalities has been proven to be associated with the severity of the bone marrow disorder. The most common abnormality of bone marrow is isochromosome (7)(q10), (i(7q)), which accounts for up to 40% of abnormalities 2. The significance of i(7q) is controversial 7, 8, 9, 10. The present report describes two male siblings with SDS, one that harbored cytogenetic abnormality of i(7q) in bone marrow and required allogeneic HCT.

## Case Presentation

### Case 1

This male infant was born at full‐term from healthy unrelated parents and had a body weight of 2356 g. Body weight was 4495 g (−3.2SD) and length was 54.3 cm (−2.4SD) at 3 months of age. Blood tests revealed anemia with a hemoglobin level of 6.3 g/dL. At 6 months of age, low levels of trypsin, lipase, amylase, and elastase‐1 were identified, indicating exocrine pancreatic insufficiency.

**Figure 1:**
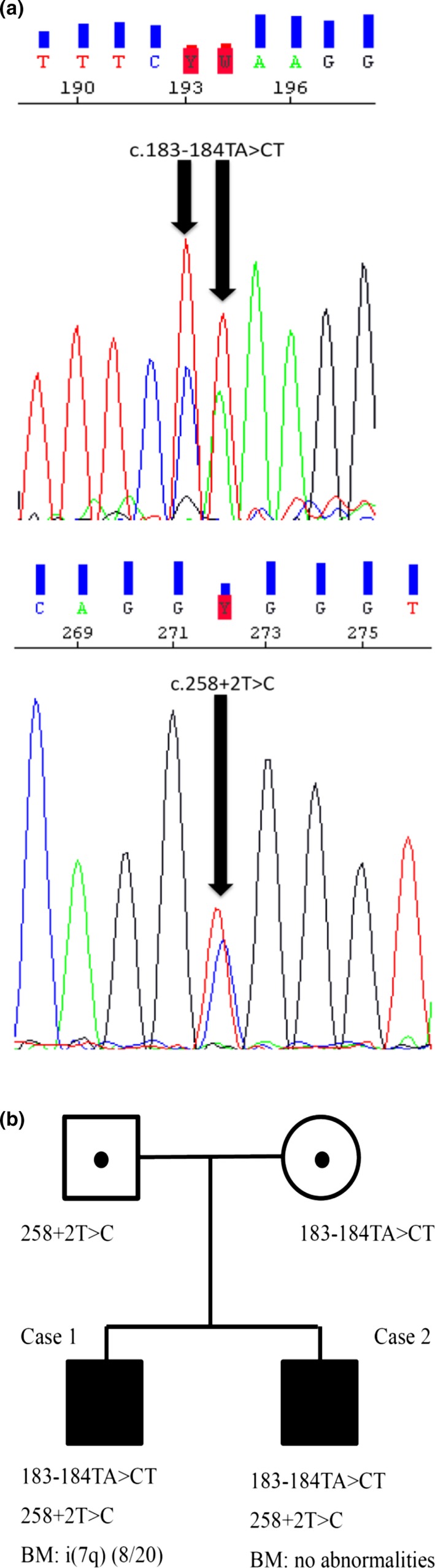
(A) Exon 2 mutations of *SBDS* gene are confirmed by PCR and the Sanger sequence. (B) The parents harbor heterozygous mutations of *SBDS* gene exon2 and two children harbor heterozygous mutation (c.183‐184TA>CT and c.258+2T>C).

We performed polymerase chain reaction (PCR) and Sanger sequencing of the SBDS gene. Total cellular DNA was extracted from peripheral blood cells with a Blood DNA Kit (Qiagen, Tokyo, Japan). Exon 2 of the SBDS gene was amplified using the primers and PCR conditions as follows: forward and reverse primers were 5′‐TTGGGGGGTAAGAAAAAGA‐3′ and 5′‐GCTTGGTTAGTCTTTCCTCC‐3′, respectively. Cycling conditions were as follows: one cycle at 94°C for 2 min; 35 cycles consisted of denature at 94°C for 1 min, annealing at 53°C for 1 min, and extension at 72°C for 1 min, and 1 cycle at 72°C for 10 min. PCR products were cleaned up using PCR purification kit (Qiagen) and sequenced with Dye Terminator method. All samples were analyzed by a ABI PRISM 310 Genetic Analyzer 4, 11, 12 (Fig. 1A). There are no mutations in other exons.

We found that the patient had compound heterozygous mutations of the SBDS gene (183‐184TA>CT and 258+2T>C) (Fig. 1B). He was diagnosed as having SDS. At the time of diagnosis, there were no chromosomal abnormalities in the bone marrow. His father harbored a heterozygous 258+2T>C mutation, and his mother harbored a heterozygous 183‐184TA>CT mutation. At the age of 3 years, he started to require frequent red blood cell and platelet transfusions. His white blood cell count ranged from 1000/mm^3^ to 3000/mm^3^ and neutrophil count from 300/mm^3^ to 500/mm^3^. G‐banding analysis of his bone marrow at the age of 3 years showed i(7q) in eight of 20 cells.

The patient underwent allogeneic bone marrow transplantation from an HLA full‐matched unrelated donor at 3 years 11 months old. The conditioning regimen consisted of antithymocyte globulin (2 mg/kg × 3 days; −18 day to −16 day), fludarabine (25 mg/m^2^; −3 day to −7 day), and melphalan (90 mg/m^2^ −3 day to −2 day), and tacrolimus and short‐term methotrexate were used for graft‐versus‐host disease prophylaxis. Engraftments of neutrophils and platelets were achieved on days 16 and 23 after HCT, respectively. Full donor chimerism of bone marrow was identified on day 31. He has not received a transfusion for 4 years after engraftment.

### Case 2

The younger brother of Case 1 was born at full‐term with a birthweight of 2402 g. A lack of weight gain was noted at the age of 4 months. Because his older brother was diagnosed as SDS, genetic screening was performed. The infant harbored the same compound heterozygous mutations of the SBDS gene as Case 1: that is, 183‐184TA>CT and 258+2T>C.

Unlike Case 1, cytogenetic abnormalities including i(7q) were not identified despite repeated bone marrow aspirations. The patient has not had severe cytopenia since birth. His white blood cell count ranged from 1500/mm^3^ to 3000/mm^3^ (neutrophil count 300–700/mm^3^), hemoglobin from 8 to 10 g/dL, and platelet count from 150,000/mm^3^ to 230,000/mm^3^. HCT has not been performed due to the mild manifestation of the disease. He is currently 5 years old.

## Discussion

Of the two male siblings with SDS, the older brother had severe cytopenia and required allogeneic HCT. Compound heterozygous mutations of 183‐184TA>CT and 258+2T>C are common mutations in Japanese SDS patients, which demonstrate heterogeneous phenotypes 11. As only the older brother (Case 1) had i(7q), it is indicated that i(7q) is associated with the development of severe cytopenia related to SDS.

Maserati et al. 7 reported two female siblings with SDS, one who demonstrated the constitutional presence of i(7q). That report noted that the patient with i(7q) died of progressive cytopenia, whereas the other sibling who was without i(7q) did not have progressive cytopenia. Cada et al. 8 suggested that SDS patients who have clones with i(7q) may have progressive bone marrow failure because all of the patients with i(7q) had severe cytopenia.

On the other hand, some reports counter the risk of myeloid malignancies in SDS patients with i(7q), indicating a good prognosis of SDS with i(7q) 9, 10. However, refractory cytopenia in SDS patients with i(7q) was described in these articles 9, 10, and the significance of i(7q) in cytopenia has not yet been clarified. The clinical courses of the siblings who harbored the same compound mutations although severe cytopenia was manifested in only the sibling with i(7q) indicated that i(7q) is associated with the progression of severe bone marrow failure in SDS patients.

Despite an intensive effort, we could not find detailed reports that showed an association of i(7q) with cytopenia in SDS. Therefore, further studies should be performed to conclude whether isochromosome (7)(q10) leads to severe cytopenia. However, a large‐scale study presents a challenge, considering the small number of patients with this disease. Despite this limitation, our report suggests that clinicians should carefully monitor the blood counts of patients with SDS with i(7q).

## Consent

Written informed consent was obtained from the parents of these patients for publication of this case report and any accompanying images. A copy of the written consent form is available for review by the Editor‐in‐Chief of this journal.

## Conflict of Interest

We have no conflict of interest.

## Authorship

YS, RT, ST, RK, MDY, SI, and SY: developed the conception of the case report. MT, KS, HG, and MDY: were involved in clinical care of the patients and obtained clinical data. JN performed polymerase chain reaction and sequencing. YS, RT, ST, and YDM: wrote the initial draft of the manuscript. MT, KS, RK, HG, JN, SI, and SY: critically reviewed and revised the manuscript. All the authors approved the final version of the manuscript.
